# Diagnostic Methods and Biomarkers in Inflammatory Bowel Disease

**DOI:** 10.3390/diagnostics15111303

**Published:** 2025-05-22

**Authors:** Andrew M. Kaz, Nanda Venu

**Affiliations:** 1 GI Section, Hospital and Specialty Medicine, VA Puget Sound Health Care System, Seattle, WA 98108, USA; 2 Department of Medicine/Gastroenterology, University of Washington School of Medicine, Seattle, WA 98195, USA; nanda.venu@commonspirit.org; 3 Center for Digestive Health, Virginia Mason Franciscan Health, Seattle, WA 98101, USA

**Keywords:** inflammatory bowel disease, biomarker, Crohn’s disease, ulcerative colitis, diagnosis, endoscopy, intestinal ultrasound, video capsule endoscopy, artificial intelligence

## Abstract

Inflammatory bowel disease (IBD) refers to a chronic inflammatory condition involving the GI tract that includes Crohn’s disease (CD) and ulcerative colitis (UC). These conditions are believed to arise in genetically predisposed individuals who develop an exaggerated immune response to the intestinal microbiota. A timely and accurate diagnosis of IBD is essential because diagnostic delays can result in intestinal damage that is irreversible, leading in some cases to intestinal dysfunction and the need for surgery. Diagnostic delays are common in cases when GI symptoms are mild and nonspecific. When IBD is suspected, the common diagnostic algorithm includes laboratory analyses, cross-sectional radiologic imaging, and endoscopy with biopsy and histological analysis. Other diagnostic biomarkers, including those found in the serum, stool, and urine, have also been evaluated in IBD. Newer artificial intelligence (AI)-based technologies are now being developed, and these will likely play an important future role in the diagnosis and management of IBD.

## 1. Introduction

Inflammatory bowel disease (IBD) refers to a group of chronic, immune-mediated inflammatory processes involving any portion of the alimentary canal, characterized by an unpredictable clinical course that can result in permanent damage to the gastrointestinal (GI) tract [1,2]. Idiopathic IBD is typically divided into ulcerative colitis and Crohn’s disease, although these conditions frequently overlap and differentiating them can be challenging [3]. Generally, the inflammation in ulcerative colitis (UC) is limited to the mucosa, starting in the rectum and extending to more proximal parts of the colon. Crohn’s disease (CD), meanwhile, is characterized by a transmural inflammatory process that can involve any portion of the GI tract and be associated with strictures, fistulas, or abscesses [2]. The etiology of IBD is complex and remains an area of active investigation but is believed to result from an aberrant host immune response to environmental factors and the intestinal microbiota in a genetically predisposed individual [1].

Historically, the incidence of IBD has been greatest amongst the Caucasian population in industrialized nations. More recently, the incidence and prevalence are increasing in newly industrialized countries, including those in Asia, Africa, and Latin America [4,5,6]. The reasons for this shift are not entirely clear but may be related in part to differences in an individual’s environment exposures and lifestyle [7]. Environment factors include exposure to pollution, quality of sanitation, and contact with chemicals such as per- and polyfluoroalkyl substances (PFASs) [8]. Commonly implicated lifestyle factors include breastfeeding (which may be protective) and consuming a highly processed diet, which is associated with an increased risk of developing IBD [9,10].

The change in IBD incidence has led to rising numbers of IBD cases globally and an associated increase in healthcare costs [11]. Complicating the increasing global burden of IBD is the fact that the diagnosis of UC and Crohn’s can be challenging as these conditions can present with non-specific GI symptoms [12]. This frequently leads to delays in diagnosis (especially with Crohn’s disease), which puts individuals at risk for complications including irreversible bowel damage [13].

With an appropriate awareness of the clinical presentation of IBD, the healthcare team has at their disposal a wide variety of diagnostic methods to deploy. These include the more traditional techniques, such as endoscopy, histological assessment, and imaging, and more novel techniques including biomarker analyses. Most of these diagnostic methods are also important tools used for managing individuals already diagnosed with IBD, playing a prominent role in assessing disease activity and planning for changes in treatment regimens. This review summarizes many of these diagnostic methods and markers, with special attention given to the use of artificial intelligence (AI) in the diagnosis and management of IBD.

## 2. Diagnosis of IBD

### 2.1. Clinical Presentation

The clinical presentation of inflammatory bowel diseases is highly variable. Individuals can present with dramatic, severe symptoms, such as hematochezia, abdominal pain, intestinal obstruction or perforation, or malnutrition. Alternatively, symptoms can be mild and non-specific, which often results in an inaccurate or delayed diagnosis [12,14]. At times, the timing or type of symptoms can provide clues to the location or subtype of IBD. UC most commonly presents with blood in the stool and diarrhea, but can also include fecal urgency, increased bowel movement frequency, tenesmus, and abdominal cramping. Extra-intestinal manifestations (EIMs) occur in 30% or more of patients with UC, and in a minority of cases, EIMs can precede the onset of GI symptoms [1]. EIMs include arthritis (most common) as well as involvement of the skin, eyes, and hepatobiliary system (primary sclerosing cholangitis) [15,16,17].

Blood in the stool is less commonly seen in Crohn’s disease unless the colon is the primary site of involvement. The clinical presentation in CD depends upon the location, severity, and chronicity of inflammation [2]. Abdominal pain, diarrhea, weight loss, and fatigue are common [18]. EIMs are also frequently seen in individuals with Crohn’s disease. Perianal disease, including fissures, ulcerations, skin tags, and fistulas, are seen in approximately 35% of those with Crohn’s, and these complications can precede the onset of GI luminal disease [19].

When IBD is suspected based on the clinical presentation, and other potential causes such as infections or medication side effects have been excluded, the diagnostic workup can proceed. This typically includes laboratory-based testing (which might demonstrate evidence of anemia or inflammation), endoscopy with biopsy and histological analysis, and cross-sectional imaging. These and other diagnostic modalities are discussed in further detail below.

### 2.2. Endoscopy

Endoscopy is a critical initial diagnostic procedure when Crohn’s disease or ulcerative colitis is suspected. Ileocolonoscopy with or without upper endoscopy (esophagogastroduodenoscopy; EGD) allows the clinician to determine the location, extent, and severity of IBD involvement. During colonoscopy, intubation of the terminal ileum is recommended, which can help differentiate CD and UC [1,20]. Upper endoscopy is often performed at the same time as the initial colonoscopy if Crohn’s disease is suspected so the proximal GI tract can be assessed for involvement.

Endoscopic findings in the colon of individuals with UC typically include friable mucosa, ulcerations, erythema, and reduction or loss of the normal luminal vascular appearance [21]. These changes normally include the rectum and extend proximally to a variable degree, involving a portion of or the entire colon in a circumferential, continuous pattern [22]. In some cases, the cecum may show isolated, patchy inflammation associated with only distal colonic disease [23]. Those with long-standing inflammation may develop abnormal appearing (or absent) colonic haustra, strictures, stenoses, or inflammatory polyps [12]. While a minority of UC patients with pancolitis might have mild inflammation of the terminal ileum (TI), often called “backwash ileitis”, Crohn’s disease should be considered in those with only distal colonic inflammation and evidence of active disease involving the TI [21].

Whereas the disease in UC is continuous, the inflammation in CD is often patchy, notable for “skip lesions” (areas of affected intestine interspersed between normal bowel). Typical endoscopic findings include aphthous and serpiginous ulcerations and edematous, nodular mucosa which yields a “cobblestone” pattern [2]. Areas containing strictures or stenoses, or fistulous openings, can sometimes be seen [24]. Special attention should be given to the perianal area during the endoscopic evaluation, which may demonstrate large skin tags, fistulous openings, or fissures.

### 2.3. Histopathology

During endoscopy, samples are collected with biopsy forceps from various segments of the colon and terminal ileum for histological analysis. Most commonly, six regions are targeted, including the TI, ascending colon, transverse colon, descending colon, sigmoid colon, and rectum. Normal appearing areas should be sampled as well as clearly affected regions [24]. Common histological findings in UC include crypt abnormalities, chronic inflammatory cells within the lamina propria, Paneth cell metaplasia, and mucosal erosions [22,25]. The European Crohn’s and Colitis Organisation (ECCO) notes a UC diagnosis is based on “widespread crypt architectural distortion, a diffuse transmucosal inflammatory infiltrate with basal plasmacytosis, eventually associated with an active component, causing cryptitis and crypt abscesses.” The group notes that basal plasmacytosis is the first and most predictive feature of UC, whereas crypt alterations might develop later; thus, normal crypt architecture does not rule out early UC [25].

In suspected cases of Crohn’s disease, biopsies are also collected from the TI and various colonic segments for microscopic evaluation. Typical histologic features of CD involving the colon include focal chronic inflammation, crypt irregularity, and granulomas. In the ileum, similar features can be seen, along with irregular villous architecture [25]. The granuloma seen in Crohn’s disease is epithelioid and located in the lamina propria and should be distinguished from similar-appearing lesions associated with crypt injury (“crypt rupture”) or non-caseating granulomas which can be associated with infectious colitis caused by organisms such as *Mycobacterium* or *Yersinia* [25,26]. If found, a non-cryptolytic granuloma can be a reliable differentiator favoring CD over UC, although the clinical utility is limited, as only approximately 15% of mucosal biopsies in those with CD will contain granulomas [25,27].

### 2.4. Imaging Tests

#### 2.4.1. Cross-Sectional Imaging

Imaging tests such as CT enterography (CTE) and MR enterography (MRE) are important diagnostic procedures for IBD. These tests overcome some of the limitations of endoscopy, in that they permit evaluation of the entire bowel, which is particularly useful in cases of suspected Crohn’s disease. Additionally, CTE and MRE are non-invasive, generally better tolerated than endoscopy, and may permit detection of extraintestinal disease [28].

Cross-sectional imaging techniques have distinct advantages and disadvantages when compared, and factors such as patient preference, local expertise, and the specific clinical indication are all pertinent when deciding upon the most appropriate test. The diagnostic accuracy of CTE and MRE are similar, although the overall accuracy may be higher with MRE [29,30]. A meta-analysis of 33 studies found a sensitivity and specificity of 93.0% and 92.8% for MRE versus 84.3% and 95.1% for CTE; these differences were not statistically significant [31]. Given the similar performance characteristics, the authors of this study concluded that use of diagnostic modalities that do not involve ionizing radiation (i.e., MRE) is preferable. Other experts agree with prioritizing MRE when possible to limit cumulative radiation exposure, emphasizing the fact that most patients with IBD will require multiple imaging tests over their lifetimes [2,12,30,32].

Cross-sectional imaging is especially beneficial in diagnosing and managing Crohn’s disease, as more than half of patients with CD will have small intestinal or other abnormalities on CT/MR despite a normal colonoscopy. The intestinal inflammation present in CD can be located proximal to the furthest extent reached during ileocolonoscopy or can be found only deep to the mucosa or within the intestinal mesentery [33]. Imaging tests can also provide important information about disease behavior or phenotype in CD, demonstrating the presence of strictures, bowel dilation, and fistulas [34]. Individuals with overt or suspected perianal Crohn’s should be evaluated with pelvic MRI, which can delineate fistulous tracts and other abnormalities [32,35].

#### 2.4.2. Intestinal Ultrasound

Intestinal ultrasound (IUS) is a non-invasive technique that is becoming more widely available and can play an important role in IBD diagnosis and management. IUS has many attractive features: not only is it non-invasive, but it is accurate, well-tolerated, preferred by patients, and allows for real-time clinical diagnosis and decision making [36,37,38]. IUS permits the user to detect bowel inflammation (characterized by bowel wall thickness > 3 mm) in both the colon and small intestine [39]. In addition to bowel inflammation, other parameters assessed by IUS include hyperemia (a measure of increased blood flow to the inflamed bowel), loss of the normal stratification of the bowel wall, and increased mesenteric fat inflammation [36,40,41]. Penetrating and stricturing complications of IBD, such as strictures and abscesses, can also be assessed [35,42].

IUS is an accurate tool that can be used at the time of IBD diagnosis and can be especially useful for detecting small bowel abnormalities in patients with symptoms that are suspicious for Crohn’s. Castiglione et al. found that IUS detected small bowel CD with a sensitivity of 94% and a specificity of 97%, with a positive predictive value (PPV) of 97% and a negative predictive value (NPV) of 94% [42]. A meta-analysis of 15 studies analyzing the accuracy of IUS for diagnosing CD reported a summary area under the receiver operating characteristic (ROC) curve of 0.94, indicative of high diagnostic accuracy [43]. Although MRE and CTE are generally used more commonly than IUS for IBD diagnosis, IUS appears to be at least as sensitive as these other modalities, with an excellent NPV that makes it appealing as an initial diagnostic test [32,41]. The 2019 ECCO-ESGAR (European Society of Gastrointestinal and Abdominal Radiology) guideline suggests the use of either MRE or IUS to assess individuals with suspected or newly diagnosed CD, noting similar diagnostic accuracy between the two imaging tests [42,44]. When comparing the utility of MRI and IUS in assessing IBD activity in patients with disease involving only the colon and terminal ileum, IUS appears to be the more accurate imaging modality, with an overall accuracy of 89% for IUS versus 73% for MRI [45].

#### 2.4.3. Video Capsule Endoscopy

A video capsule endoscopy (VCE) test involves ingestion of a swallowable capsule containing a camera that records images of the small bowel (SB) during transit. VCE can be an important diagnostic procedure for CD, where SB involvement is common. Nearly a third of individuals with CD have isolated small intestinal involvement, which can be challenging to diagnose, and VCE allows direct visualization of the small bowel mucosa where even subtle abnormalities can be detected [46,47,48]. Both European and U.S. guidelines suggest the use of VCE (also known as small bowel capsule endoscopy; SBCE) for evaluation of suspected CD [44,49,50].

The diagnostic yield of VCE compares favorably to other imaging techniques. One meta-analysis of 12 studies comparing VCE to small bowel radiography, CTE, and MRE found VCE to be superior in cases of suspected CD. The yield of VCE versus small bowel radiograph was 52% versus 16%, and VCE versus CTE was 68% versus 21% [51]. Another meta-analysis of 13 studies found similar overall diagnostic yields comparing VCE and MRE in CD, while VCE was superior to MRE for detection of proximal SB disease [52].

Some studies have questioned the specificity of VCE in cases of suspected Crohn’s disease. A study by Solem et al. noted similar sensitivities between VCE, CTE, ileocolonoscopy, and small-bowel follow-through (SBFT) for detecting active small bowel CD, but a lower specificity for VCE (53%) compared to the other tests [53]. The authors concluded that VCE should be reserved for cases of suspected CD despite a negative evaluation with cross-sectional imaging and ileocolonoscopy. Combining a biomarker, such as fecal calprotectin, with VCE has been proposed to improve the specificity of VCE for a CD diagnosis [54].

Other groups have recommended VCE in patients with suspected CD and negative ileocolonoscopy findings as the initial diagnostic test, assuming there are no obstructive symptoms or evidence of stricturing disease [50,55]. VCE has a high negative predictive value (96%) and thus appears to be useful for excluding cases of suspected CD [56].

Limitations of small bowel capsule endoscopy include the uncommon risk of the capsule becoming entrapped in the intestine, which can require surgical removal, and the lack of means to obtain tissue for diagnosis [49]. Known small bowel strictures are a contraindication for VCE examination. The rate of capsule retention based on 10 studies of individuals with suspected CD varied between 0 and 5.4%, and in cases of confirmed CD, the retention rate ranged from 0 to 13.2% [57]. Thus, many would recommend a small bowel radiological exam or placement of a patency capsule (a dissolvable capsule given prior to VCE to ensure successful passage through the intestine) before VCE [57,58]. If VCE detects abnormal appearing areas in the small intestine indicative of CD, device-assisted enteroscopy (including single or double-balloon enteroscopy) can be performed to obtain a tissue diagnosis [59].

### 2.5. Biomarkers for IBD Diagnosis and Management

#### 2.5.1. Serologic Markers

A variety of serological biomarkers are routinely obtained at the time IBD is suspected or first diagnosed. Although the results from biomarker assessment can be suggestive of UC or Crohn’s disease, no marker is entirely sensitive or specific for IBD [44]. Hemoglobin and serum albumin levels may be reduced at the time of diagnosis [60]. Elevations in serum C-reactive protein (CRP) and erythrocyte sedimentation rate (ESR) often reflect active CD but tend to be less useful in those with UC. These markers often correlate with disease activity, can be used to predict response to advanced medical therapies, and may have a role predicting the risk for colectomy in those with UC [61,62]. Notably, ESR and CRP levels can be normal in up to 40% of IBD patients even when there is clear evidence of endoscopic inflammation, which limits their utility in monitoring disease activity [63,64].

Antibodies against microbial antigens and several autoantibodies have previously been discovered and evaluated for their role in diagnosing IBD or discriminating UC from CD [49]. For example, 70% of individuals with UC may have detectable serum perinuclear antineutrophil cytoplasmic antibodies (pANCAs), and the combination of detectable pANCAs with negative anti-*Saccharomyces cerevisiae* antibodies (ASCA) has been proposed as a diagnostic panel specific for UC [60,65]. One study determined that a pANCA+/ASCA- result could diagnose UC with a sensitivity of 78% and specificity of 67%, whereas a pANCA-/ASCA+ result could diagnose CD with a sensitivity of 67% and specificity of 78% [66]. Other antibody markers, including anti-outer-membrane porin C (OmpC) and anti-Cbir1, can be detected in about 50% of individuals with CD, and only 5–10% of individuals with UC or healthy controls [67,68]. Overall, the accuracy of these tests is limited, and they are ineffective at differentiating Crohn’s disease from UC [44,69]. Additionally, although more than 200 single-nucleotide polymorphisms (SNPs) associated with IBD have been identified, none of the common variants allow for a definitive diagnosis of IBD to be made [70].

#### 2.5.2. Fecal Biomarkers

Several neutrophil-derived proteins detectable in stool have been evaluated as markers of intestinal inflammation, including fecal calprotectin (FC), lactoferrin, and elastase [63,65]. Of these, FC appears to be the most sensitive and generally correlates well with inflammation noted during endoscopy [49]. FC can also be useful as an initial test to discriminate IBD from functional diarrheal disorders such as irritable bowel syndrome (IBS) [71], with one meta-analysis reporting good diagnostic precision in distinguishing IBD from non-IBD using an FC cutoff of 100 μg/g [72]. Fecal calprotectin cannot differentiate between various causes of intestinal inflammation, including infections and adverse effects from medications [73]. Table 1 summarizes the conventional diagnostic tests for IBD.

#### 2.5.3. Novel Biomarkers

In addition to the more commonly utilized biomarkers described above, other markers for IBD have been detected in the stool, urine, and serum of individuals with Crohn’s disease and UC [29]. Serum nitric oxide (NO) levels have been shown to vary between individuals with CD, UC, and normal controls, and NO levels can increase during UC exacerbations [74]. Serum cytokines such as TNF-α, which may promote inflammation in IBD and are a target of medical therapy in some IBD patients, may be elevated in individuals with IBD. One study found the median concentration of serum TNF-α to be 390-fold higher in IBD patients compared to healthy controls, and that TNF-α levels increased by 1.7-fold in active versus inactive UC [75]. Other cytokines, such as suppression of tumorigenicity 2 (ST2) and TNF-α- induced protein 6 (TNFAIP6), may also be increased in those with UC and CD compared to those without IBD [76,77].

Oncostatin M (OSM) is a cytokine that has been found to be elevated in tissues of those with IBD and likely plays a role in promoting chronic inflammation [78]. At the time of diagnosis, individuals with both UC and CD demonstrate elevated OSM levels compared to healthy controls. OSM levels were also able to predict post-operative disease occurrence and poor response to the anti-TNF class of medications [79,80]. Another class of biomarkers that may have utility in diagnosing UC include the anti-integrin αvβ6 antibodies, which may bind and disrupt an important protein which maintains epithelial barriers. A study by Kuwada et al. found 92% of those with UC and only 5% of healthy controls had detectable antibodies against integrin αvβ6, resulting in a diagnostic sensitivity and specificity of 92% and 95%, respectively [81].

Other biomarkers detectable in the stool include S100A12 and Lipocalin-2/neutrophil gelatinase-associated lipocalin (NGAL). A study in children with IBD found S100A12 levels up to 20 times higher in IBD patients compared to healthy controls, with a sensitivity of 96% and specificity of 92% for IBD diagnosis at a cutoff value of 10 mg/kg [82]. NGAL levels, meanwhile, were found to be more than 10 times that of normal controls in IBD patients, with a sensitivity and specificity for IBD diagnosis of 95% and 96%, respectively [83]. In a prospective study by Ling Lundstrom et al., the group examined fecal specimens from individuals with newly diagnosed CD, UC, symptomatic controls (SCs), and healthy controls (HCs). They found several markers to be elevated in those with IBD, with myeloperoxidase (MPO; AUC 0.85) and FC (AUC 0.85) demonstrating the best overall accuracy in distinguishing IBD from SCs. The diagnostic accuracy was best when MPO and FC were combined, and levels of FC, MPO, and human neutrophil lipocalin (HNL) were predictive of an aggressive disease phenotype [84]. Additional fecal biomarkers for IBD are listed in Table 2.

Urine biomarkers, while less established compared to other types of biomarkers, have also been associated with inflammation in IBD. Matrix metalloproteinases (MMPs) might play a role in T-cell-mediated gut injury and may be elevated in the urine of those with UC and Crohn’s disease [85]. One study analyzed 95 urine samples from known or suspected IBD patients and found that levels of MMP-2 and MMP-9/NGAL were independent predictors of CD and UC [86]. Another study evaluated prostaglandin E-major urinary metabolite (PGE-MUM) in urine specimens from 104 UC and 39 functional GI disorder patients using a rapid chemiluminescent enzyme immunoassay. They found PGE-MUM levels were elevated more in the UC group (*p* < 0.0001), and while PGE-MUM was inferior to fecal calprotectin for detecting histologic remission, it was still a potential non-invasive marker for determining endoscopic activity in pediatric UC [87].

MicroRNAs (miRNA) are small, noncoding RNA molecules that are involved in RNA silencing and post-transcriptional regulation of gene expression. They normally circulate in blood and other body fluids, and alterations in their expression appear to play a role in various diseases [29]. Altered expression of numerous miRNAs is seen in individuals with IBD, and these miRNAs may play a role in various inflammatory processes [88]. One group noted that circulating levels of miR-223 were higher in those with IBD versus controls, and miR-223 levels correlated with disease activity [89]. Another study showed that circulating levels of miR-320a correlated closely with endoscopic disease activity in individuals with UC and CD [90]. A recent meta-analysis including 15 studies involving 937 IBD patients and 707 controls found 22 miRNAs and two miRNA panels suitable for analysis. The overall sensitivity and specificity for IBD diagnosis were 0.80 (95% CI: 0.79–0.82) and 0.84 (95% CI: 0.82–0.86), respectively, with an area under the curve (AUC) of 0.89 [91]. The authors concluded that these miRNAs are credible diagnostic markers with moderate diagnostic accuracy for IBD.

Long non-coding RNAs (lncRNAs) represent another class of nucleic acids that can be detected in tissue or serum samples from individuals with IBD and may be potential biomarkers for IBD. These RNA molecules are at least 200 nucleotides in length and regulate gene expression and translation and are known to be involved in a multitude of biological processes, including inflammation [92]. At least eight different lncRNAs have demonstrated altered expression levels in studies aimed at diagnosing individuals with IBD [92]. Wang et al. determined that serum levels of lncRNAs FIF9-AS1 could discriminate between those with CD and healthy controls (AUC of 0.81) and also those with UC versus healthy controls (AUC of 0.87). Also, two additional lncRNAs (DIO3OS and LINC01272) could differentiate those with CD from healthy controls (AUC of 0.79 and 0.89, respectively) and those with UC from healthy controls (AUC of 0.65 and 0.78, respectively) [93].

A multitude of studies have incorporated metabolomic analyses of tissue, plasma, or feces collected from patients with IBD and found alterations in metabolites that can be used to diagnose IBD or differentiate CD from UC [94]. These studies are driven in part by the knowledge that there are differences in the microbiome of IBD patients compared to healthy controls, and these differences can be detected in the stool [88,95,96]. For example, one study using liquid chromatography-mass spectrometry (LC-MS) to analyze stool from 155 IBD and 65 non-IBD individuals found increased levels of sphingolipids and bile acids and decreased levels of triacylglycerol and tetrapyrrole in IBD patients [96]. Another study utilized ultra-performance liquid chromatography time-of-flight mass spectrometry (UPLC-TOF-MS) to analyze the stool from 32 UC patients and 23 healthy controls and discovered downregulation of certain secondary bile acids (including lithocholic and deoxycholic acids) and upregulation of other bile acids (such as taurocholic and cholic acids) in the UC patients [97].

The analysis of differential protein and lipid profiles (proteomics and lipidomics) has also been evaluated as a means to diagnose, monitor, and differentiate the subtypes of IBD [98]. A study by Manfredi et al. using a shotgun proteomic approach to analyze serum from 28 IBD patients and 17 controls found altered levels of the proteins KAIN, PRCC, and GELS in CD patients versus healthy controls and altered levels of LPPRC, SURF4, and CHADL in UC patients versus healthy controls. They also found a total of 11 differentially regulated fatty acids in CD, two differentially regulated fatty acids in UC, and three down-regulated lipids in both CD and UC patients (eicosapentaenoic acid (EPA), docosahexaenoic acid (DHA), and linoleic acid (LA)) [99].

Specific analyses of gut microbiome components focusing on either certain bacterial genes or species can be used to classify or diagnose IBD. Studies comparing the microbiota between IBD and non-IBD cases highlight the fact that alterations in the gut microbiomes (“dysbiosis”) frequently exist in those with IBD [100]. One study of 2045 non-IBD and IBD fecal samples from four European countries found dysbiosis was greater in CD versus UC, with reduced microbial diversity and increased microbial instability in CD samples, and that eight groups of bacteria could be used to distinguish CD from non-CD [101]. The authors concluded these microbiome subtypes could be useful to distinguish between UC and CD when the diagnosis is in doubt. Serrano-Gomez et al. used a metagenomic approach to evaluate stool from European and U.S. cohorts, including more than 300 individuals with either IBD or healthy controls. They found taxonomic profiles could differentiate CD from non-CD with an AUC of 0.938 and UC from healthy controls with an AUC of 0.646 [102]. In another study by Zheng et al., they used metagenomic data from 5979 fecal samples from individuals with or without IBD and chose a panel of bacterial species to develop diagnostics tests for both UC and CD. Both models had AUCs of > 0.90 in the discovery cohort. They subsequently developed PCR tests for individual bacterial species and found them to be more accurate than FC for discriminating IBD from controls [103].

Overall, while the novel biomarkers described above appear to discriminate between individuals with IBD and healthy controls and may provide interesting insights in the pathogenesis of IBD, none are routinely used for clinical diagnosis at this time. Further validation of these markers in larger and more diverse populations is required before they can be included in diagnostic algorithms. These biomarkers are summarized in Table 2.

**Table 2:** Novel biomarkers for diagnosis of IBD.

Biomarker	Notes	References
Serological markers NO, TNF-α, IL-10, ST2, TNFAIP6, OSM, anti- integrin αvβ6	Elevated in IBD vs. non-IBD May increase during IBD flares	[74,75,76,77,78,81]
Fecal markers S100A2, NGAL, MMP-9, MPO, HNL	Elevated in IBD vs. non-IBD May increase during IBD flares	[82,83,84,104,105]
Urine markers MMP-2, MMP-9, MMP-9-NGAL, PGE-MUM	Elevated in IBD vs. non-IBD	[86,87]
MicroRNA markers miR-233, miR320a, miR-16, miR-21, miR-223, others	Differential expression in IBD vs. non-IBD	[89,90,91,106]
Long non-coding RNA markers FIF9-AS1, DIO3O3, LINC01272, others	Differential expression in IBD vs. non-IBD	[92,93]
Metabolomic markers sphingolipids, bile acids, triacylglycerol, tetrapyrrole, others	Differential expression in IBD vs. non-IBD	[94,96,97]
Proteomic/lipidomic markers KAIN, PRCC, GELS, EPA, DHA, LPPRC, SURF4, CHADL, others	Differential expression in IBD vs. non-IBD	[98,99]
Microbiome markers taxonomic profiles, specific genera/species (e.g. *Faecalibacterium* *prausnitzii*), others	Can distinguish UC from CD and IBD from non-IBD	[100,101,102,103]

Abbreviations: NO, nitric oxide; CD, Crohn’s disease; UC, ulcerative colitis; TNF-α, tumor necrosis factor-alpha; IBD, inflammatory bowel disease; IL-10, interleukin 10; ST2, suppression of tumorigenicity 2; TNFAIP6, TNF-α- induced protein 6; NGAL, lipocalin-2/neutrophil gelatinase-associated lipocalin; MMP-9, matrix metalloprotease-9; MPO; myeloperoxidase; MMP-2, matrix metalloprotease-2; EPA, eicosapentaenoic acid; DHA, docosahexaenoic acid.

## 3. Cost Effectiveness of Diagnostic Tests

There are limited studies comparing the cost-effectiveness of the various diagnostic tests for IBD. Regional or international differences in diagnostic algorithms are primarily driven by local expertise, availability of equipment, and payer coverage. Most of the more recent research involving cost effectiveness is focused on biomarkers such as fecal calprotectin. A Canadian study evaluated the cost effectiveness of FC plus serum CRP/ESR versus CRP/ESR alone in a hypothetical primary care setting and found that FC was expected to cost more than blood tests alone but overall was likely to be cost effective and reduce the time to IBD diagnosis [107]. An Italian study evaluated the cost-effectiveness of MRI, IUS, ileocolonoscopy, and VCE in a model of hypothetical suspected Crohn’s disease cases. The authors found that the cost depended on the pre-test probability of CD, with ileocolonoscopy plus IUS being the least expensive initial diagnostic strategy [108]. Another large multicenter UK study compared the accuracy and cost effectiveness of MRE and IUS in 284 patients newly diagnosed with Crohn’s disease and found the cost effectiveness to be similar [109].

## 4. Role of Artificial Intelligence in the Diagnosis and Management of Inflammatory Bowel Disease

Artificial intelligence (AI) holds significant promise for transforming the care of patients with IBD in the future. Much like the human brain, AI systems “learn” by acquiring information, identifying patterns, and observing outcomes of the decisions made from the available data [110]. However, AI can analyze and learn from larger, more complex datasets with unprecedented speed and accuracy compared to the human brain. The abundant availability of electronic medical data and advancements in computing power has enabled the growth of AI in IBD care. The expansion of complex pattern-recognition data analytics, or “machine learning” (ML), is driving AI innovation in IBD diagnostics, disease activity monitoring, and therapeutic response prediction [110].

Machine learning techniques such as support vector machines (SVMs), decision tree models (including regression trees and random forests), and artificial neural networks (ANNs) are commonly used in AI systems. These techniques enable the analysis of both structured and unstructured data from digital medical records, including radiological imaging, endoscopic imaging, laboratory test results, and text from clinical documentation. This capability has already led to numerous advancements in the field.

Upper endoscopy and colonoscopy are fundamental tools for diagnosing IBD, monitoring disease activity, and assessing therapeutic response. However, significant interobserver variability exists in the interpretation of endoscopic disease severity indices for ulcerative colitis and Crohn’s disease [111]. AI may help mitigate this interobserver variability. Several studies have demonstrated the ability of AI-enabled endoscopy to predict disease severity, correlate with histological disease activity, and accurately forecast therapeutic response. For instance, AI-powered systems have been shown to improve the standardization of endoscopic scoring systems, such as the Mayo Endoscopic Score for UC and the Simple Endoscopic Score for Crohn’s Disease (SES-CD) [112,113,114,115]. Recently, new computer-assisted detection (CAD) devices have been used to analyze mucosal architectural features (e.g., crypts and pericryptal capillaries) in real time. In a recent study of 58 UC patients, this new AI technology successfully predicted UC histological remission with high accuracy (86%) as compared to standard endoscopic scoring [116]. In another study, a deep neural network was developed which evaluated endoscopic images from UC patients and was able to predict endoscopic remission with 90% accuracy and histologic remission with 93% accuracy without requiring endoscopic biopsy or histologic analysis [113].

Artificial intelligence has also been evaluated for colonic dysplasia detection in IBD. Dysplastic lesions consist of precancerous mucosal alterations that can be difficult to detect due to their flat nature and indistinct borders. Maeda et al. used an AI-based detection system during endoscopy, which placed “bounding boxes” around presumed dysplastic lesions. These areas were targeted for endoscopic biopsy, and low-grade dysplasia was later confirmed histologically in all cases [117].

AI applications have also extended to video capsule endoscopy (VCE), an important, minimally invasive diagnostic tool for evaluating small bowel lesions in CD. Studies have reported lesion detection accuracies in individuals with CD ranging from 92.4% to 97.1% using AI-driven models [118,119]. These systems can precisely distinguish ulcers, strictures, and other mucosal abnormalities. Furthermore, AI assistance can significantly reduce the time required to interpret VCE studies, making this diagnostic modality a more efficient and reliable test.

Cross-sectional radiological imaging, including CT, MRI, and intestinal ultrasound, plays a critical role in IBD diagnosis and in assessing IBD complications such as strictures, abscesses, and fistulas. AI-driven tools may help standardize radiological reporting and reduce interobserver variability. For example, convolutional neural networks (CNNs), a type of deep learning algorithm, have been used to detect and quantify bowel wall thickening, a hallmark of active IBD, with high precision [28]. These advancements may enhance the accuracy of imaging-based assessments and support more consistent clinical decision making. Other recent studies exploring the feasibility of AI-assisted cross-sectional imaging in identifying IBD disease activity have shown AI can mimic the multiscale assessment and reporting of radiologists, leading to enhanced diagnostic accuracy and increased productivity [120,121].

Existing and emerging biomarkers, many of which were discussed previously, are increasingly used for diagnosis, monitoring, and predicting therapeutic response in IBD. AI technologies are highly suited to leverage large volumes of genetic, molecular, and “omics” data, and machine-learning algorithms can enable precision medicine—tailoring treatments to the individual needs of each patient. For instance, studies have demonstrated the utility of ML models in predicting response to biologic therapies based on transcriptomic and proteomic data [122,123,124,125]. These studies demonstrate the potential of AI to analyze clinical, genetic, gut microbiome, and inflammatory marker data to predict relapse risk and response to various treatment strategies. By distinguishing treatment responders and non-responders, AI technologies may help identify patients most likely to benefit from specific treatments, minimizing trial-and-error approaches and improving clinical outcomes.

Although the data on cost effectiveness in AI are limited, it is expected that AI can reduce costs associated with managing IBD by improving diagnostic accuracy and timeliness and predicting treatment response, leading to reductions in healthcare costs. AI models enhance diagnostic accuracy using genomic data and predict response to medical therapies, optimizing expensive treatment choices [126]. An accurate prediction of an individual’s estimated response to a particular IBD therapy could reduce the time to achieve disease remission and minimize switching between medical therapies, eventually leading to cost savings. In terms of procedures, the use of AI may lead to reduced numbers of procedures and associated costs. One study demonstrated that AI-assisted endoscopy could be used to classify colon polyps as “low risk”, and if these polyps were left in vivo and not submitted to the pathology lab for analysis, colonoscopy costs could be reduced by 10.9%, saving an estimated $85.2 million annually in the U.S. [127]. Additionally, AI-enhanced digital health remote monitoring tools have shown success in reducing outpatient visits and hospital admissions without compromising care quality. Monitoring applications include symptom reporting tools and promoting self-care and healthy behaviors and can reduce healthcare utilization while improving patient satisfaction [128]. Overall, AI integration offers significant potential for improving outcomes while lowering costs in IBD care.

The application of AI in IBD presents significant ethical considerations, including concerns related to algorithmic bias, lack of transparency, data privacy, and disparities in access. Predictive models may underperform in minority populations if trained on non-representative datasets, potentially leading to unequitable and/or inaccurate clinical predictions [129]. The opaque decision-making processes of many AI algorithms can undermine clinician confidence and complicate informed consent. Additionally, the integration of AI-based remote monitoring tools raises substantial data protection concerns, highlighted by the Google Alphabet-owned DeepMind NHS data-sharing controversy. This collaboration between a large, powerful organization and a public health entity yielded valuable lessons regarding openness, privacy, and the use of population-based datasets by commercial enterprises [130]. Access to AI technologies remains uneven, with advanced tools more readily available in high-resource settings. These gaps might exacerbate existing healthcare disparities, as seen with AI-driven diabetic retinopathy screening in low-resource populations. While there is a presumed benefit in extending screening to additional populations, there are concerns about the generalizability of the data derived from the training population [131]. These examples underscore the necessity for rigorous validation, transparent algorithm development, equitable data use, and comprehensive regulatory oversight to ethically integrate AI into IBD clinical practice [132,133,134].

Although direct care delivery to IBD patients by AI remains a distant prospect, its impact is already evident. AI is poised to influence nearly every aspect of IBD management, from diagnosis, disease assessment, and therapeutic decision making to biomarker discovery and patient communication. As AI technologies continue to evolve, interdisciplinary collaboration among clinicians, researchers, data scientists, and ethicists will be essential to ensure that these tools are developed and implemented responsibly.

In conclusion, AI represents a transformative force in IBD care, offering the potential to enhance diagnostic accuracy, personalize treatments, and improve clinical efficiency. Continued advancements in AI research, coupled with thoughtful consideration of ethical implications, will be pivotal in realizing its full potential.

## 5. Conclusions

IBD is a chronic inflammatory condition of the GI tract with no known cure that traditionally includes CD and UC. Diagnosing IBD is the crucial first step before initiating treatments which are often life long and involve immune system suppression. In most cases, diagnostic evaluation precedes in individuals with suspected IBD with laboratory evaluation, cross-sectional imaging, and ileocolonoscopy. A tissue diagnosis, typically achieved with a biopsy during endoscopy, is currently the diagnostic gold standard and generally required to finalize the diagnosis. Early diagnosis of IBD is crucial in order to initiate appropriate medical therapy as soon as possible and reduce the likelihood of intestinal damage and dysfunction [135]. The diagnosis can be challenging in cases where symptoms are mild and non-specific, which increases the risk of irreversible damage to the intestines. Novel IBD biomarkers, potentially combined with clinical data analysis using AI technologies, could lead to improvements in the speed and accuracy of diagnosis.

## Figures and Tables

**Table 1:** Overview of conventional diagnostic methods in IBD.

Method	Advantages	Disadvantages
Endoscopy and histology	Direct visualization of mucosa Tissue acquisition for diagnosis	Invasive, inconvenient Cannot easily access most of the SB
Imaging		
CTE	Non-invasive Good accuracy; permits SB eval Info on extra-intestinal findings	Radiation exposure
MRE	Non-invasive Good accuracy; permits SB eval Info on extra-intestinal findings No radiation exposure	Possibly limited availability of equipment/local expertise
IUS	Non-invasive Good accuracy, high NPV Real-time info for clinician	Operator dependent Less accurate with presence of excessive gas and for proximal gut
VCE	Minimally invasive Very good diagnostic yield, high NPV Evaluation of entire small bowel	Possibly lower specificity vs. other tests No tissue acquisition for diagnosis Risk of capsule entrapment
Serologic tests		
ESR, CRP,	Non-invasive Can mirror disease activity	Nonspecific Frequently normal even during IBD flare
pANCA, ASCA, anti-OmpC, anti-Cbir1	Non-invasive	Limited sensitivity and specificity
Fecal tests		
Fecal calprotectin	Non-invasive Correlates well with disease activity	Nonspecific

Abbreviations: SB, small bowel; CTE, computed tomographic enterography; MRE, magnetic resonance enterography; IUS, intestinal ultrasound; NPV, negative predictive value; VCE, video capsule endoscopy; ESR, erythrocyte sedimentation rate; CRP, C-reactive protein; pANCA, perinuclear antineutrophil cytoplasmic antibodies; ASCA, anti-*Saccharomyces cerevisiae* antibodies; anti-OmpC, anti-outer-membrane porin C; anti-Cbir1, anti-flagellin antibody.
